# An enhanced password authentication scheme for session initiation protocol with perfect forward secrecy

**DOI:** 10.1371/journal.pone.0194072

**Published:** 2018-03-16

**Authors:** Shuming Qiu, Guoai Xu, Haseeb Ahmad, Yanhui Guo

**Affiliations:** 1 School of CyberSpace Security, Beijing University of Posts and Telecommunications, Beijing, 100876, China; 2 Elementary Educational College, Jiangxi Normal University, Nanchang, 330022, China; 3 Department of Computer Science, National Textile University, Faisalabad, 37610, Pakistan; King Saud University, SAUDI ARABIA

## Abstract

The Session Initiation Protocol (SIP) is an extensive and esteemed communication protocol employed to regulate signaling as well as for controlling multimedia communication sessions. Recently, Kumari et al. proposed an improved smart card based authentication scheme for SIP based on Farash’s scheme. Farash claimed that his protocol is resistant against various known attacks. But, we observe some accountable flaws in Farash’s protocol. We point out that Farash’s protocol is prone to key-compromise impersonation attack and is unable to provide pre-verification in the smart card, efficient password change and perfect forward secrecy. To overcome these limitations, in this paper we present an enhanced authentication mechanism based on Kumari et al.’s scheme. We prove that the proposed protocol not only overcomes the issues in Farash’s scheme, but it can also resist against all known attacks. We also provide the security analysis of the proposed scheme with the help of widespread AVISPA (Automated Validation of Internet Security Protocols and Applications) software. At last, comparing with the earlier proposals in terms of security and efficiency, we conclude that the proposed protocol is efficient and more secure.

## 1 Introduction

The Session Initiation Protocol (SIP) is an important and popular communications protocol for signaling and controlling multimedia communication sessions in applications including Internet telephony for voice and video calls, private IP telephone systems, as well as instant messaging over Internet Protocol (IP) networks [1, 2]. Up to now, SIP has gained the attention of extensive scholastic community.

The first authentication scheme for SIP based on hyper text transfer protocol (HTTP) digest authentication can be traced back to 1999 proposed by Franks et al. [3]. In 2005, Yang et al. [4] pointed out that the scheme of Franks et al. [3] cannot resist the off-line password guessing attack and the server impersonation attack. Subsequently, Yang et al. [4] presented an new scheme to cope with the aforementioned issue in [3]. However, Huang et al. [5] proved that Yang et al.’s [4] scheme cannot resist the stolen-verifier, the off-line password guessing and the Denning-Sacco attacks [6], and is not suitable for power constraint devices because of the high computational cost. In 2005, in order to improve Yang et al.’s [4] scheme, Durlanik and Sogukpinar [7] proposed an efficient and secure authentication scheme for SIP using the Elliptic Curve Cryptography (ECC). It is known that ECC could provide the same security with a smaller key size comparing with the other traditional Public Key Cryptography. Subsequently, numerous one-factor, two-factor and three factor authentication schemes have been proposed for SIP using ECC, RSA, Hash function or Chaotic theory, etc [7–25].

### 1.1 Related works

Recently, Zhang et al. [26] pointed out that the existing protocols for SIP require the SIP server maintaining a password or verification table, which makes these protocols vulnerable to stolen-verifier attack, server spoofing attack, insider attack, and password-guessing attack. To address these issues, Zhang et al. proposed a new two-factor authentication protocol for SIP by using smart cards to avoid maintenance of password tables at the SIP server.

Later, Zhang et al. [27] showed that their scheme [26] is prone to impersonation attack problem. To remedy this problem, the authors proposed a much improved protocol based on Zhang et al.’s protocol [26] by using smart card. However, Farash [28] pointed out that Zhang et al. protocol [27] is still insecure against the impersonation attack. Thereupon, Farash proposed an improved protocol by making a slight change in Zhang et al. protocol [27]. However, Lu et al. [29] analyzed the security of Farash’s [28] scheme and pointed out that the enhanced scheme presented by Farash et al. [28] has still some security vulnerabilities, including key-compromise impersonation attack, off-line guessing attack and lack of anonymity, pre-verification. Afterwards, Lu et al. designed a preserving anonymous authentication protocol to remedy the security limitations of Farash’s scheme. The authors showed that their scheme is resistance to all known attacks besides those attacks existed in Farash’s scheme. But subsequently, Kumari [30] showed that an adversary is able to calculate the user’s identity and password once the adversary obtains the datum of user’s smart card in Lu et al. [29]’s scheme. Thus, Kumari [30] claimed that Lu et al.’s scheme does not adhere to two-factor security criterion. Besides, the author also pointed out that the key agreement procedure of Lu et al. [29]’s scheme cannot culminate to achieve the intended aim of authenticated key agreement. On the other hand, in order to eliminate the drawbacks of Zhang et al. [26]’s scheme, Irshad et al. [31] also developed an enhancement SIP authentication scheme only using a single round-trip in 2005. But, Arshad et al. [32] found that the improvement of Irshad et al. [31] was also susceptible to the user impersonation attack and further proposed their improved scheme regarding performance and security analyses. However, the modified scheme of Arshad et al. [32] was demonstrated to be lacking user anonymity and mutual authentication and susceptible to the key-compromise impersonation attack by Lu et al. [33]. In 2014, Jiang et al. [34] also observed that Zhang et al.’s scheme [26] was prone to the user impersonation attack and made a few modifications to enable more secure than the original design. Azrour et al. [35] showed that Jiang et al.’s protocol suffers from server impersonation attack.

In 2014, Tu et al. [36] also proved that Zhang et al. [26]’s scheme is vulnerable to user impersonation attack. Furthermore, Tu et al. [36] proposed an enhanced protocol to improve the security. However, Farash [37] pointed out that Tu et al.’s scheme is still vulnerable to server impersonation attack and proposed an improvement in Tu et al.’s scheme. In 2015, Chaudhry et al. [38] also showed that Tu et al.’s scheme [36] is vulnerable to server impersonation, replay and denial of services attacks as well as lacking user anonymity. Moreover, Chaudhry et al. [38] also analyzed that Farash’s improvement [37] on Tu et al.’s scheme [36] is lacking user anonymity and is also vulnerable to replay attack. Thereupon, Chaudhry et al. [38] proposed an anonymous authenticated key agreement scheme while claiming that it is more secure and suitable for all lightweight environments. Recently, Kumari et al. [39] also analyzed Farash’s protocol [37] and showed that it is vulnerable to user impersonation attack, password guessing attack, session-specific temporary information leakage attack and lacks to provide user anonymity. Furthermore, Kumari et al. [39] proposed an improved protocol, and showed that their protocol is not only robust against all known attacks, but is also lightweight as compared to Farash’s protocol [37]. From the above analysis, one can observes that most of these protocols have still some security loopholes and not really reach the security of the authentication protocol. Accordingly, it is still a challenging academic topic to design a more secure and efficient authentication and key agreement protocol for SIP.

### 1.2 Contribution of this paper

**Fig 1:**
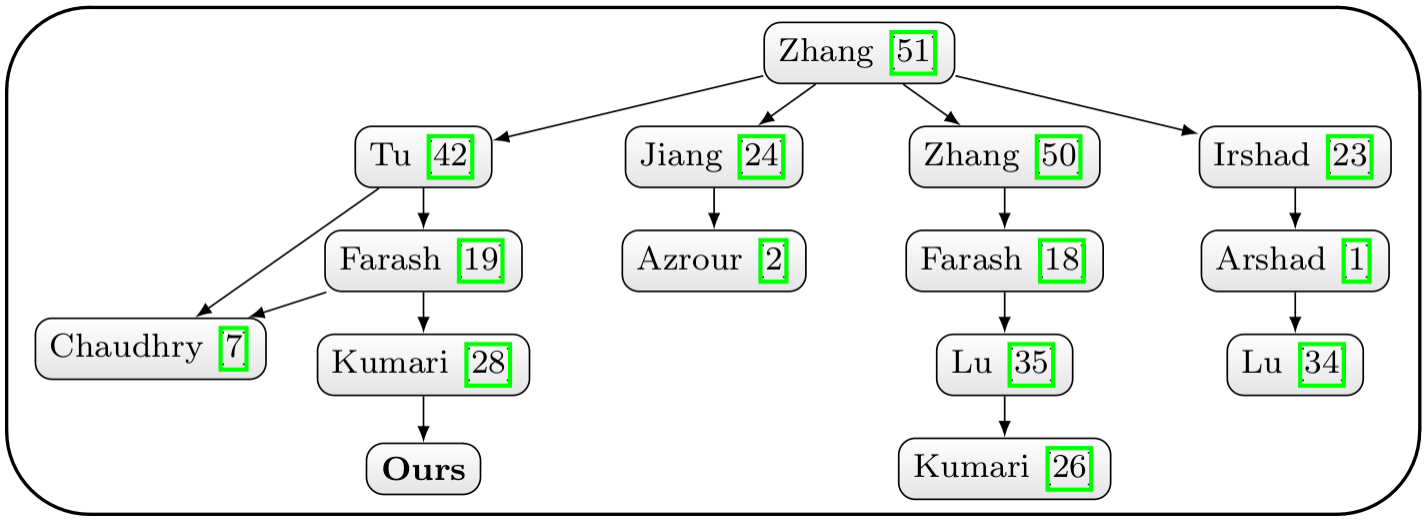
Positional relation of the proposed scheme.

The positional relation of the proposed scheme and related researches are depicted in Fig 1. The contributions of this paper are listed as follows:

- We concentrate on analyzing the security of Kumari et al. [39]’s authentication scheme for SIP, and point out that Kumari et al. [39]’s scheme fails to provide pre-verification, local password change in smart card and perfect forward secrecy, is also susceptible to key-compromise impersonation attack.
- To overcome aforementioned limitations, we propose an improved scheme while maintaining the benefits of the original schemes at the cost of slight increase in the computation consumptions by employing “Fuzzy-Verifier” [40]. Besides, we prove that our scheme provides various security features including perfect forward secrecy and resistance against key-compromise impersonation attack, etc.
- We use AVISPA tool to prove that proposed scheme satisfies the mutual authentication and session key secrecy.
- We provide security and performance comparisons with various relevant schemes. It illustrates that the proposed scheme is efficient and more secure than the prevalent schemes.

### 1.3 Organization of this paper

The remainder of this paper is organized as follows: Section “Preliminaries” introduces some notations, associated difficult problems based on ECC and adversary model used in this paper. The review and cryptanalysis of Kumari et al. [39]’s scheme is detailed in Section “Review of Kumari et al.’s scheme” and Section “Cryptanalysis of Kumari et al.’s scheme”, respectively. Section “The enhanced scheme for SIP” provides our proposed scheme. Section “Security analysis of the enhanced scheme” and Section “Formal security validation using AVISPA tool” highlight an informal and formal security analysis of our scheme, respectively. The performance and functionality comparison is presented in Section “Comparative analysis of performance”. At last, we provide concluding remarks in Section “Conclusion”.

## 2 Preliminaries

In this section, we describe some notations and the definitions of one-way hash function and hard problems related with the Elliptic Curve Cryptography(ECC) and the capacities of the adversary in this paper. Some notations used in this paper are listed in Table 1.

**Table 1:** Notations and abbreviations.

Symbol	Description
*S*	Server
*U*	Patient/User
*ID*	Identity of *U*
*PW*	Password of *U*
*c*_*u*_, *a*_*u*_	Random numbers of *U*
*k*_*s*_	Secret key of *S*
*b*, *c*_*s*_	Random numbers of *S*
||	The string concatenation operation
⊕	The bitwise XOR operation
A	Malicious adversary
*h*(⋅)	Collision free one-way hash function
→	An insecure channel
⇒	A secure channel
*sk*	Session key between *U* and *S*

### 2.1 Intractable problems

**Definition 1** (Collision-resistant one-way hash function) A secure one-way hash function *h*(⋅): {0, 1}* → {0, 1}^*n*^ takes an arbitrary length binary string *x* ∈ {0, 1}* as an input, and outputs a binary string *y* = *h*(*x*) ∈ {0, 1}^*n*^. A cryptographic hash function *h*(⋅) satisfies the following properties.

1. It is hard to find the the input *x* ∈ {0, 1}* in polynomial time for given *y* ∈ {0, 1}^*n*^;
2. It is hard to find *x*′ ∈ {0, 1}* such that *x*′ ≠ *x* and *h*(*x*) = *h*(*x*′);
3. It is hard to find a pair (*x*, *x*′) ∈ {0, 1}* such that *h*(*x*) = *h*(*x*′), where *x*′ ≠ *x*.

In ECC, the elliptic curve equation is defined as the form of *E*_*p*_(*a*, *b*): *y*^2^ = *x*^3^ + *ax* + *b*(mod *p*) over a finite field *F*_*p*_, where *a*, *b* ∈ *F*_*p*_ and 4*a*^3^ + 27*b* ≠ 0(mod *p*).

**Definition 2** (ECDLP) For given generator *P* and *Q* = *mP* in *E*_*p*_(*a*, *b*), where *m* is randomly selected from *F*_*p*_ and *p* is sufficiently large prime, it is computationally hard by a probabilistic polynomial time (PPT) adversary A to calculate the secret value *m* ∈ *F*_*p*_ such that *Q* = *mP*.

**Definition 3** (ECCDHP) For given points *mP*, *nP* ∈ *E*_*p*_(*a*, *b*), computing *mnP* is computationally infeasible by a probabilistic polynomial time (PPT) adversary A.

### 2.2 Adversary model

Throughout this paper, according to [40–43], the capacities of the adversary A are summarized as follows:

1. The adversary A has the capability to extract all parameters stored in smart card utilizing the power analysis method [41, 42].
2. The adversary A is able to control the open communication channel completely, i.e. he can intercept, modify, delete, block, and resend the messages over the open channel.
3. The adversary A can list all pairs of (*ID*_*i*_, *PW*_*i*_) from $\left(D_{PW},D_{ID}\right)$ in a polynomial time, where $D_{PW}$ and $D_{ID}$ denote the space of passwords and the space of identities, respectively.
4. The adversary A can either intercept the password of the user via malicious device or extract the parameters from smart card, but not both.
5. While evaluating forward secrecy, the adversary A can obtain server’s private key or comprise of the user’s password.
6. When it comes to key-compromise impersonation attack, we assume that A knows the long-term private key of server.

## 3 Review of Kumari et al.’s scheme

### 3.1 System setup phase

The server *S* chooses an elliptic curve *E* over the finite field *F*_*q*_ and an additive group *G* of order *p* with *P* as generator, a one-way hash function *h*(⋅), a secret key $k_{s}\in Z_{p}^{*}$ computes its public key *Q* = *kP*. At last, *S* publishes its public parameters {*E*(*F*_*q*_), *P*, *p*, *Q*, *h*(⋅)}, and keeps *k*_*s*_ as its long-term private key.

### 3.2 Registration phase

In this phase, the user *U* is registered as a legal user by executing the following steps over the secure channel:

**Step 1:** User *U* selects his identity *ID*, password *PW* and a random number $a_{u}\in Z_{p}^{*}$. Then, he computes *VPW* = *h*(*ID*||*PW*||*a*_*u*_) and sends the registration request message {*ID*, *VPW*} to server *S*
**Step 2:** After receiving the request message {*ID*, *VPW*}, *S* calculates *r*_*u*_ = (*VPW* + *h*(*ID*||*k*_*s*_))*P*, and stores *r*_*u*_ in a new smart card *SC*. Also, *S* issues *SC* = {*r*_*u*_, *Q* = *k*_*s*_*P*, *h*(⋅)} to *U*
**Step 3:** Upon receiving the new smart card *SC*, *U* inserts *a*_*u*_ in *SC*. Finally, *SC* = {*r*_*u*_, *Q* = *k*_*s*_*P*, *a*_*u*_, *h*(⋅)} and *U* is thus registered as a legal user.

### 3.3 Login and mutual authentication phase

In this phase, user *U* establishes the session key with server *S* as follows:

**Step 1:** *U* inserts his smart card *SC* to a card reader and inputs his identity *ID* and password *PW*.
**Step 2:** *U* selects a random number $b\in Z_{p}^{*}$, and computes *bP*, *V* = *bQ*, *W*_*u*_ = *b*(*r*_*u*_ − *VPW* ⋅ *P*). *U* further calculates *f*_*u*_ = *ID* ⊕ *V*_*x*_, *z*_*u*_ = *h*(*ID*||*bP*||*V*_*y*_||*W*_*u*_), where *V*_*x*_, *V*_*y*_ are *x*^*th*^, *y*^*th*^ components of *V*, respectively. At last, *U* sends the login request message {*f*_*u*_, *bP*, *z*_*u*_} to *S*.
**Step 3:** After receiving the request message {*f*_*u*_, *bP*, *z*_*u*_}, *S* computes *V* = *k*_*s*_*Q*. Subsequently, *S* computes *ID* = *f*_*u*_ ⊕ *V*_*x*_ and further calculates $W_{u}^{*}=h(ID||k_{s})bP,z_{u}^{*}=h(ID||bP||V_{y}\left|\right|W_{u}^{*})$. *S* then checks whether $z_{u}^{*}\overset{?}{=}z_{u}$. If it holds, *S* chooses a random number $c\in Z_{p}^{*}$ and calculates $sk=h(W_{u}^{*}\left||bP||V||c||ID\right)$, *Auth*_*s*_ = *h*(*c*||*sk*). Afterwards, *S* sends the challenge request message {*c*, *Auth*_*s*_} to *u*.
**Step 4:** After receiving the challenge message {*c*, *Auth*_*s*_}, *U* calculates $sk=h(W_{u}||bP||Q||V||c||ID),Auth_{s}^{*}=h\left(c||sk\right)$. *U* then checks whether $Auth_{s}^{*}\overset{?}{=}Auth_{s}$. If it holds, *U* calculates *Auth*_*u*_ = *h*(*ID*||*c* + 1||*sk*) and sends the response message {*Auth*_*u*_} to *S*.
**Step 5:** Once receiving the response message {*Auth*_*u*_}, *S* computes $Auth_{u}^{*}=h(ID||c+1||sk)$. *U* then verifies whether $Auth_{u}^{*}\overset{?}{=}Auth_{u}$. If $Auth_{u}^{*}=Auth_{u}$, *S* believes that it has successfully established the session key *sk* with *U*.

### 3.4 Password changing phase

In this phase, *U* can change his password by interacting with the server *S*. After *U* establishes the session key *sk* with *S*, *U* changes his password by performing the following steps:

**Step 1:** User *U* selects his new password *PW*^*new*^ and two random numbers $a_{u}^{new},e\in Z_{p}^{*}$. Subsequently, he computes *VPW*^*new*^ = *h*(*ID*||*PW*^*new*^||*a*^*new*^) and then calculates *m*_*u*_ = *Enc*_*sk*_(*ID*||*e*||*VPW*^*new*^||*h*(*ID*||*e*||*VPW*^*new*^)). At last, *U* send the request message {*m*_*u*_, *e*} to server *S*.
**Step 2:** After receiving the request message {*m*_*u*_, *e*}, *S* computes *Dec*_*sk*_(*m*_*u*_) = *ID*||*e*||*VPW*^*new*^||*h*(*ID*||*e*||*VPW*^*new*^). Subsequently, *S* verifies the validity of *h*(*ID*||*e*||*VPW*^*new*^). If it passes the validity test, afterwards *S* calculates $r_{u}^{new}=(VPW^{new}+h(ID||k_{s}))P,m_{s}=Enc_{sk}(r_{u}^{new}||h(ID||e+1||r_{u}^{new}))$. *S* then sends response message {*m*_*s*_} to *U*.
**Step 3:** Upon getting the message {*m*_*s*_}, *U* decrypts *m*_*s*_ and obtains $r_{u}^{new},h(ID||e+1||r_{u}^{new})$. Subsequently, *U* verifies the validity of $h(ID||e+1||r_{u}^{new})$. If it passes the validity test, *U* replaces $r_{u}^{new},a_{u}^{new}$ with *r*_*u*_, *a*_*u*_, respectively.

## 4 Cryptanalysis of Kumari et al.’s scheme

Kumari et al. [39] claimed that their scheme can resist many known attacks. However, we explain minutely that the scheme of Kumari et al. not only fails to provide pre-verification in smart card, perfect forward secrecy and efficient password changing, but also fails to resist key-compromise impersonation attack in the following subsections. Actually, the above functions are fundamental and crucial to authentication scheme for session initiation protocol. Accordingly, these imply that their scheme is still unsuitable for the practical session initiation protocol.

### 4.1 Pre-verification in smart card

When a user inputs her/his password and identity, if the smart card verifies their correctness, implies that respective protocol can provide pre-verification in smart card. But, Kumari et al.’s scheme is not providing such mechanism.

In the login phase of Kumari et al.’s scheme, the smart card is unable to provide any verification for the password and identity information of user because there is no verified information in smart card. If the user inputs the wrong password and identity or an adversary A performs this step, the smart card fails to check this problem. Until the server finds the incorrectness of the login, the session will not be terminated. In this case, it increases computational cost of server. Consequently, Kumari et al.’s scheme is unable to provide the pre-verification in smart card.

### 4.2 Key-compromise impersonation attack

Let us consider a scenario that when the long-term private key of server *S* is compromised, an adversary A can certainly impersonate the legal server of being legitimate user, but if A is not impersonated as the legal user by the corresponding server, we say that this protocol can resist key-compromise impersonation attack. It is a pity that Kumari et al.’s scheme is unable to withstand this attack. Now, let’s execute the following steps to attack their scheme.

**Step 1:** Firstly, the adversary A gets some useful information {*r*_*u*_, *kP*, *a*_*u*_} stored in smart card utilizing the side-channel attack [41]. A then captures the login request message {*f*_*u*_, *bP*, *z*_*u*_} of user. If the long-term private key *k* of *S* is revealed to A, A computes *V* = *k*(*bP*), and further calculates the real identity *ID* = *f*_*u*_ ⊕ *v*_*x*_. As an illegal user, A randomly selects $b^{\prime }\in Z_{p}^{*}$ and computes *V*′ = *b*′(*kP*), $w_{u}^{\prime }=b^{\prime }(r_{u}-h(ID||PW||a_{u}))P=b^{\prime }h\left(ID||k\right)P,f_{u}^{\prime }=ID⊕V_{x}^{\prime },z_{u}^{\prime }=h(ID||b^{\prime }P||V_{y}\left|\right|w_{u}^{\prime })$. Subsequently, the adversary A sends the forged request message $\left\{f_{u}^{\prime },b^{\prime }P,z_{u}^{\prime }\right\}$ to *S*.
**Step 2:** On receiving the request message, *S* then computes *V*′ = *k*(*b*′*P*), $ID=f_{u}^{\prime }⊕V_{x}^{\prime },w_{u}^{*}=h\left(ID||k\right)b^{\prime }P,z_{u}^{*}=h(ID||b^{\prime }P||V_{y}^{\prime }||w{*}_{u})$ and checks the correctness of $z_{u}^{\prime }$. Obviously, $z_{u}^{*}=z_{u}^{\prime }$. This infers that the illegal user A is successfully authenticated by server *S*. *S* further chooses a random number $c\in Z_{p}^{*}$ and calculates $sk=h(w_{u}^{*}\left|\right|b^{\prime }P||kP||V^{\prime }\left||c||ID\right)$, *Auth*_*s*_ = *h*(*c*||*sk*). Finally, the server *S* returns the message {*c*, *Auth*_*s*_} to A.
**Step 3:** On receiving the challenge message from the server, A computes $sk^{\prime }=h(w_{u}^{\prime }\left|\right|b^{\prime }P||kP||V^{\prime }||c||ID),Auth_{s}^{*}=h(c||sk^{\prime })$ and verifies whether $Auth_{s}\overset{?}{=}Auth_{s}^{*}$. If it holds, then A calculates $Auth_{u}^{\prime }=h(ID||c+1||sk^{\prime })$ and sends the response message $\left\{Auth_{u}^{\prime }\right\}$ to *S*.
**Step 4:** Upon getting the response message, *S* computes $Auth_{u}^{*}=h(ID||c+1||sk)$ and checks whether $Auth_{u}^{*}=Auth_{u}^{\prime }$. We know that it is obvious. Therefore, the server *S* undoubtedly believes that it has successfully established the session key *sk* with the legal user. Actually, the server suffers from the key-compromise impersonation attack.

Accordingly, we infer that Kumari et al.’s scheme fails to resist key-compromise impersonation attack.

### 4.3 Perfect forward secrecy

In case, when the long-term private key *k* is compromised to the adversary A, A will execute the following steps to attack Kumari et al.’s scheme.

**Step 1:** A intercepts the login request message {*f*_*u*_, *bP*, *z*_*u*_} of user *S*. Afterwards, A computes *V* = *k*(*bP*) and obtains {*V*_*x*_, *V*_*y*_}.
**Step 2:** A gets *ID* = *f*_*u*_ ⊕ *V*_*x*_ and further computes $w_{u}^{*}=h\left(ID||k\right)bP$.
**Step 3:** A captures the challenge request message {*c*, *Auth*_*s*_} of server *S* and calculates
  $$\begin{array}{c} sk=h(w_{u}^{*}||bP||V||c||ID). \end{array}$$
  Afterwards, the adversary A obtains the current session key *sk* when the long-term private key *k* is revealed to A, and thus the whole session is completely exposed to A.

Therefore, Kumari et al.’s scheme fails to provide the perfect forward secrecy.

### 4.4 Efficient password changing

In the password changing phase of Kumari et al.’s scheme, if the user *U* wants to change her/his password, she/he must firstly establish the session key with the server. In this way the communication and computational overhead is increased to a large extent.

## 5 The enhanced scheme for SIP

**Fig 2:**
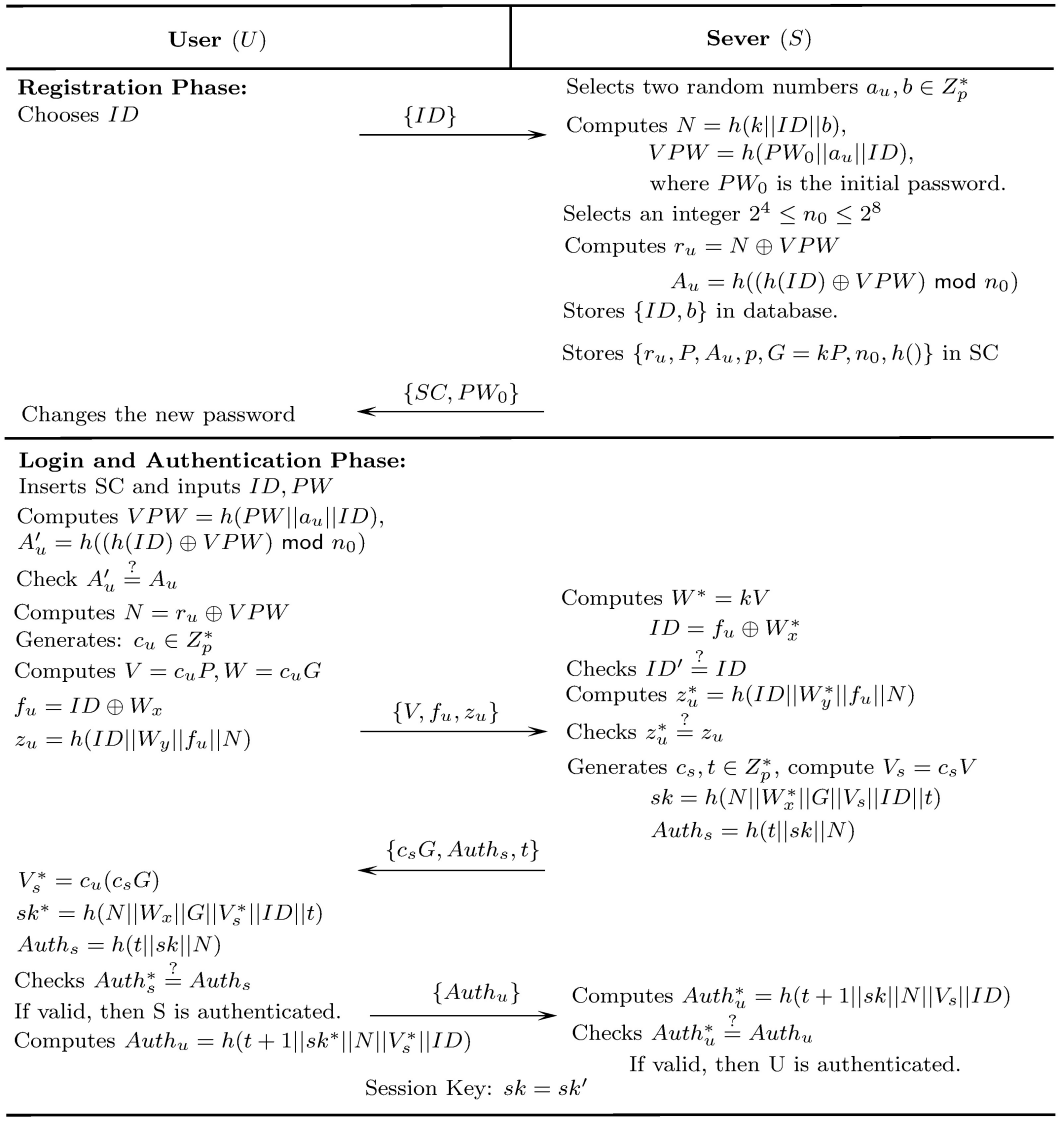
Registration and authentication phase of our scheme.

In this section, we present an improved scheme based on the Kumari et al.’s scheme. Meanwhile, our proposed scheme not only overcomes the limitations of Kumari et al.’s scheme but also achieves mutual authentication and resists against various known attacks. Specifically, we employ public-key primitive to intrinsically protect the identity of the user and provide perfect forward secrecy. In registration phase, the server *S* generates a random nonce *b* to prevent the long-term private key of *S* from being compromised. In the password changing phase, the smart card *SC* can provide the function of the local password change. The proposed scheme is comprised of four phases, i.e., system initialization, registration, login-authentication and password change. The registration and login-authentication phases are depicted in Fig 2.

### 5.1 System initialization phase

In this phase, the server *S* selects an elliptic curve *E* over the finite field *F*_*p*_, a random number $k\in Z_{p}^{*}$ and a one-way hash function *h*(⋅). *S* then computes *G* = *kP* as the public key of *S*. Finally, the server *S* publishes the parameters {*E*, *P*, *G*, *h*(⋅)}, while maintains *k*_*s*_ as the long-term private key of *S*.

### 5.2 Registration phase

**Step 1.** The user *U* chooses an identity *ID*.
**Step 2.** *U* ⇒ *S*: {*ID*}.
**Step 3.** After receiving the registration message from *U*, *S* chooses two random numbers *a*_*u*_, $b\in Z_{p}^{*}$ and calculates *N* = *h*(*k*||*ID*||*b*), *VPW* = *h*(*PW*_0_||*a*_*u*_||*ID*), where *PW*_0_ is the initial password. *S* further computes *r*_*u*_ = *N* ⊕ *VPW* and *A*_*u*_ = *h*((*h*(*ID*) ⊕ *VPW*) mod *n*_0_), where *n*_0_ is an integer and 2^4^ ≤ *n*_0_ ≤ 2^8^. Subsequently, *S* stores {*ID*, *b*} in its database.
**Step 4.** *S* ⇒ *U*: {*SC*, *PW*_0_}, where the smart card *SC* contains {*r*_*u*_, *P*, *a*_*u*_, *A*_*u*_, *p*, *G* = *kP*, *n*_0_, *h*(⋅)}.
**Step 5.** On receiving the smart card *SC* from *S*, the user *U* should immediately change the initial password during password update phase.

### 5.3 Login and mutual authentication phase

Once the patient *U* registers to the server successfully, he can send the login request to the server *S* when he wants to enjoy the service as follows:

**Step 1.** *U* inserts the smart card *SC* into a card reader and inputs *ID*, *PW*.
**Step 2.** *SC* calculates *VPW* = *h*(*PW*||*a*_*u*_||*ID*), and then computes $A_{u}^{\prime }=h\left(\left(h\left(ID\right)⊕VPW\right)\text{mod}\;n_{0}\right)$. Then *SC* checks the correctness of $A_{u}^{\prime }$ by comparing the value of *A*_*u*_ sorted in *SC*. If $A_{u}^{\prime }=A_{u}$, it shows that *ID*, *PW* are valid. Otherwise, the session is terminated.
**Step 3.** *SC* continues computing *N* = *r*_*u*_ ⊕ *VPW* and chooses a random number $c_{u}\in Z_{p}^{*}$, and then computes *V* = *c*_*u*_*P*, *W* = *c*_*u*_*G*, *f*_*u*_ = *ID* ⊕ *W*_*x*_, *z*_*u*_ = *h*(*ID*||*W*_*y*_||*f*_*u*_||*N*), where *W*_*x*_, *W*_*y*_ are *x*^*th*^, *y*^*th*^ components of *W*, respectively.
**Step 4.** *U* → *S*: {*V*, *f*_*u*_, *z*_*u*_}.
**Step 5.** After obtaining {*V*, *f*_*u*_, *z*_*u*_}, *S* calculates W* = *kV*, $ID=f_{u}⊕W_{x}^{*}$ and checks $ID_{i}^{\prime }\overset{?}{=}ID_{i}$ by searching database list. If these are not equal, *S* judges that the input password is wrong. As the wrong attempts exceed the threshold (such as 8), *S* forms a judgement that the smart card is usurped by some attacker. What’s more, *S* locks the smart card until *U* re-registers. Otherwise, *S* computes $z_{u}^{*}=h(ID||W_{y}^{*}\left|\right|f_{u}\left||N\right)$ and verifies $z_{u}^{*}\overset{?}{=}z_{u}$. If it is not found valid, *S* exits the session and counts a number *T* = 1. Alongwith, *S* suspends the card until *U* re-registers when *T* exceeds some threshold value. Otherwise, *S* generates a random number *c*_*s*_, $t\in Z_{p}^{*}$ and computes *V*_*s*_ = *c*_*s*_*V*, $sk=h(N||W_{x}^{*}\left||G|\right|V_{s}\left||ID||t\right)$, *Auth*_*s*_ = *h*(*t*||*sk*||*N*).
**Step 6.** *S* → *U*: {*c*_*s*_*G*, *Auth*_*s*_, *t*}.
**Step 7.** On receiving the message {*C*_*s*_*G*, *Auth*_*s*_, *t*}, *U* computes $V_{s}^{*}=c_{u}\left(c_{s}G\right)$,
  $$\begin{array}{c} sk^{*}=h(N||W_{x}\left||G|\right|V_{s}^{*}||ID||t), \end{array}$$
  $$\begin{array}{c} Auth_{s}^{*}=h(t||sk^{*}||N), \end{array}$$
  and checks whether $Auth_{s}^{*}\overset{?}{=}Auth_{s}$ If these are not equal, the session is terminated. Otherwise, *S* is authenticated by *U* and *U* accepts the session key sk*. Afterwards, *U* computes $Auth_{u}=h(t+1||sk^{*}\left||N|\right|V_{s}^{*}\left||ID\right)$, and sends {*Auth*_*u*_} to *S*.
**Step 8.** *U* → *S*: {*Auth*_*u*_}.
**Step 9.** After receiving the challenge message {*Auth*_*u*_}, *S* computes $Auth_{u}^{*}=h(t+1||sk||N||V_{s}\left||ID\right)$ and checks whether $Auth_{u}^{*}\overset{?}{=}Auth_{u}$. If it is found valid, then *U* is authenticated.
**Step 10.** Finally, both the patient *U* and the server *S* agree on a common session key *sk* = sk*.

### 5.4 Password update phase

This phase is incorporated to facilitate the user to change her/his password at will for which *U* and *SC* can execute the following steps:

**Step 1.** Firstly, *U* inserts the smart card into the card reader. *U* then inputs *ID*′, *PW*′ and a new password *PW*^*new*^.
**Step 2.** The smart card *SC* calculates *VPW*′ = *h*(*PW*||*a*_*u*_||*ID*), and then computes $A_{u}^{\prime }=h\left(\left(h\left(ID_{i}\right)⊕VPW\right)\text{mod}\;n_{0}\right).$ Subsequently, *SC* verifies whether $A_{u}^{\prime }=A_{u}$. If these are not equal, *SC* rejects *U* to change the password.
**Step 3.** Otherwise, *SC* generates a random number $a_{u}^{new}$ and calculates $VPW^{new}=h(PW^{new}\left|\right|a_{u}^{new}||ID),r_{u}^{new}=VPW⊕VPW^{new}⊕r_{u},A_{u}^{new}=h(\left(h\left(ID\right)⊕VPW^{new}\text{mod}\;n_{0}\right).$

Finally, *SC* stores $a_{u}^{new},r_{u}^{new},A_{u}^{new}$ in place of *a*_*u*_, *r*_*u*_, *A*_*u*_ in smart card, respectively.

## 6 Security analysis of the enhanced scheme

In this part, we prove that the proposed scheme is secure against the attacks found overlooked by Kumari et al. Besides, we show that the proposed scheme also takes care common security features. To facilitate the discussion, we also adopt the attack model proposed by Kumari et al. and the adversary model, that is, an adversary A can completely monitor the open communication channel, therefore, is able to insert, delete or modify any messages among correspondents. Moreover, A has the ability to obtain all useful information of the smart card by the side-channel attack [41]. When it comes to key-compromise impersonation attack and perfect forward secrecy, the long-term private key *k*_*s*_ is revealed to A.

### 6.1 User anonymity and user un-traceability

In this enhanced scheme, on one hand, there is no identity notations transmitted in the open channel or stored in smart card. On the other hand, suppose that the adversary A captures the messages {*V*, *f*_*u*_, *z*_*u*_}, {*c*_*s*_*G*, *Auth*_*s*_, *t*} and {*Auth*_*u*_} from the public channel. But in order to obtain the user *U*’s identity *ID*, A needs to know *W*_*x*_, which is not available since *W*_*x*_ is computed using the random number *c*_*u*_. Moreover, A cannot guess the correct identity, since, {*N*, *VPW*} are also not available. Further, even if A obtains the smart card of *U* and extracts the information in *SC*, A cannot recover the identity of *U* since *ID* is protected by one-way hash function and modulo operator. In process of login and authentication, A has no ability to trace the user’s identity, since, every transmitted message is different and does not reveal any location information about user. Therefore, the user anonymity and user un-traceability are ensured by the proposed scheme.

### 6.2 Privileged insider attack

In the registration phase, user *U* only submits *ID* to the server *S*. *S* subsequently sets an initial password *PW*_0_ for *U*. After receiving the smart card and *PW*_0_, *U* immediately changes the password that *U* knows only. Therefore, no privileged insider can access and compute user’s password, that is, the proposed scheme resists privileged insider attack.

### 6.3 Pre-verification in the smart card

In the login phase of Kumari et al.’s scheme, the smart card is inability to provide any verification for the identity and password of any user increases the burden on the server. While in our login phase, the smart card checks whether $A_{u}^{\prime }\overset{?}{=}A_{u}$ after inputting *ID*, *PW*. If it is found valid, *SC* sends the request message to *S*. Otherwise, it defers the session until the correct password and identity are entered. This implies that our method saves the computational and communication costs when there exists incorrect input or an illegal user. Consequently, the pre-verification is successfully provided by the proposed scheme.

### 6.4 Key-compromise impersonation attack

In our scheme, although the secret key *k* of the server *S* is compromised by the adversary A, A cannot impersonate the legal user *U* to cheat *S*. Because, the adversary A cannot know the random number *b* of *S* or the correct {*ID*, *PW*}, therefore, he is unable to compute the correct value of *N* though the information in smart card is extracted. Thus, A cannot calculate the correct request message {*V*, *f*_*u*_, *z*_*u*_} and cannot be authenticated by *S*. Consequently, our scheme is able to resist the key-compromise impersonation attack.

### 6.5 Server impersonation attack

Because, *k* is a long-term private key and *b* is also a random secret value of server *S*, therefore, the adversary A cannot recover W* = *kV*, *ID* = *f*_*u*_ ⊕ W*, *N* = *h*(*k*||*ID*||*b*) and is not able to forge $sk=h(N||W_{x}^{*}\left||G|\right|V_{s}\left||ID||t\right)$, *Auth*_*s*_ = *h*(*t*||*sk*||*N*). Thus, A is unable to impersonate the server *S* to the user *U*.

### 6.6 Off/On-line password guessing attack

In the proposed scheme, the adversary A cannot guess the correct identity and password of *U* even if it extracts the information {*r*_*u*_, *A*_*u*_, *G*, *n*_*o*_} in *SC*. If A guesses a pair of *ID* and *PW*, it shows that the equation $A_{u}^{\prime }\overset{?}{=}A_{u}$ must be satisfied. But according to “fuzzy-verifier” [40], A still cannot be sure if the *ID*′ and *PW*′ are the correct *ID* and *PW*, respectively. A only guesses the correct value by launching the on-line guessing to server *S*. But the number space of the *ID*′ and *PW*′ is large enough to be immune to the on-line guessing attack, therefore, the smart card *SC* remains suspended until *U* re-registers once the wrong login times exceeds the the fixed threshold. Therefore, the proposed scheme can withstand the off/on-line password guessing attack.

### 6.7 Replay attack

Suppose that A has captured all the communication messages {{*V*, *f*_*u*_, *z*_*u*_}, {*c*_*s*_*G*, *Auth*_*s*_, *t*}, {*M*_*i*_}} through open channel and tried to replay them to *U* or *S*. However, the proposed scheme takes advantage of some random numbers {*c*_*u*_, *c*_*s*_, *t*} that remain different in every session to prevent replay attack. In the process of communication, after receiving the request/challenge message, both the user and the server can immediately verify the validity of the random number everytime if A replays the communication message. Therefore, the replay attack is prevented by the proposed scheme.

### 6.8 Session-specific temporary information attack

In the proposed scheme, if the random numbers *c*_*u*_, *c*_*s*_, *t* are compromised, then the adversary A can calculate *W* = *c*_*u*_*G* and further computes *W*_*x*_. A captures the transmitted messages {*V*, *f*_*u*_, *z*_*u*_, *c*_*s*_*G*, *t*}. Afterwards, A computes *ID* = *f*_*u*_ ⊕ *W*_*x*_, *V*_*s*_ = *c*_*s*_*V*. But in order to obtain the session key *sk* = *h*(*N*||*W*_*x*_||*G*||*V*_*s*_||*ID*||*t*), A must have ability to know the value of *N* that is not available, since, *N* is protected by the private *k* and the random number *b* of server *S*. Implies, A still can not calculate the session key *sk*, although, the random numbers {*c*_*u*_, *c*_*s*_, *t*} are compromised. Therefore, the proposed protocol is secured against the session-specific temporary information attack.

### 6.9 Man-in-the-middle attack

Suppose that an adversary A intercepts the login request message {*V*, *f*_*u*_, *z*_*u*_} and the information stored in smart card. In order to launch the man-in-middle attack, A needs to compute $\left\{V^{*},f_{u}^{*},z_{u}^{*}\right\}$ for sending to server *S*. Although, A chooses a random $c_{u}^{*}$, still A cannot know the value of *N* and the real identity *ID*, therefore, he can not compute $f_{u}^{*}$ and $z_{u}^{*}$. On the other hand, even if he intercepts the challenge message {*c*_*s*_*G*, *Auth*_*s*_, *t*}, A still can not compute the forged message $\left\{c_{s}^{*}G,Auth_{s}^{*},t^{*}\right\}$ as he does not know the values of {*N*, *ID*}. Without knowing the server’s private key *k* and random number *b*, computation of *N* is computationally infeasible for the adversary A. Thus, the attacker A does not have any ability to modify the login request message or the challenge message. As a result, our scheme also resists the man-in-the-middle attack.

### 6.10 Mutual authentication

In the proposed scheme, *S* firstly checks the validity of *ID*. Afterwards, *S* authenticates *U* by verifying whether $z_{u}^{*}=z_{u}$ and checking whether $Auth_{u}^{*}=Auth_{u}$, respectively. On the other hand, *U* authenticates *S* by testing whether $Auth_{s}^{*}=Auth_{s}$. Consequently, our proposed scheme provides mutual authentication.

### 6.11 Perfect forward secrecy

When it comes to the forward secrecy, we assume that the private key *k* of *S* is compromised and that the adversary A obtains the sensitive datum {*r*_*u*_, *A*_*u*_, *G*} stored in smart card and the transmitted message {*V*, *f*_*u*_, *z*_*u*_}. A can compute *W* = *kV* and calculates *ID* = *f*_*u*_ ⊕ *W*_*x*_. But in order to calculate the previous session key *sk* = *h*(*N*||*W*_*x*_||*G*||*V*_*s*_||*ID*||*t*), A must know *c*_*u*_ or *c*_*s*_. However, it is impossible for A to obtain *c*_*u*_ from *V* or *c*_*s*_ from *c*_*s*_*G* and calculate *c*_*u*_*c*_*s*_*G* due to the intractability of *ECDLP* and *ECCDHP*. Thus, even by obtaining the private key *k* of server *S* and the smart card, the adversary A is still unable to calculate the session key *sk*. As a result, the proposed scheme provides perfect forward secrecy.

### 6.12 Efficient password changing

In the proposed protocol, if the user *U* wants change her/his password, *U* only needs to interact with the smart card *SC* to perform some operators. In this phase, the server *S* is not involved in the process of password changing. Therefore, our proposed protocol is efficient in password changing phase.

## 7 Formal security validation using AVISPA tool

**Fig 3:**
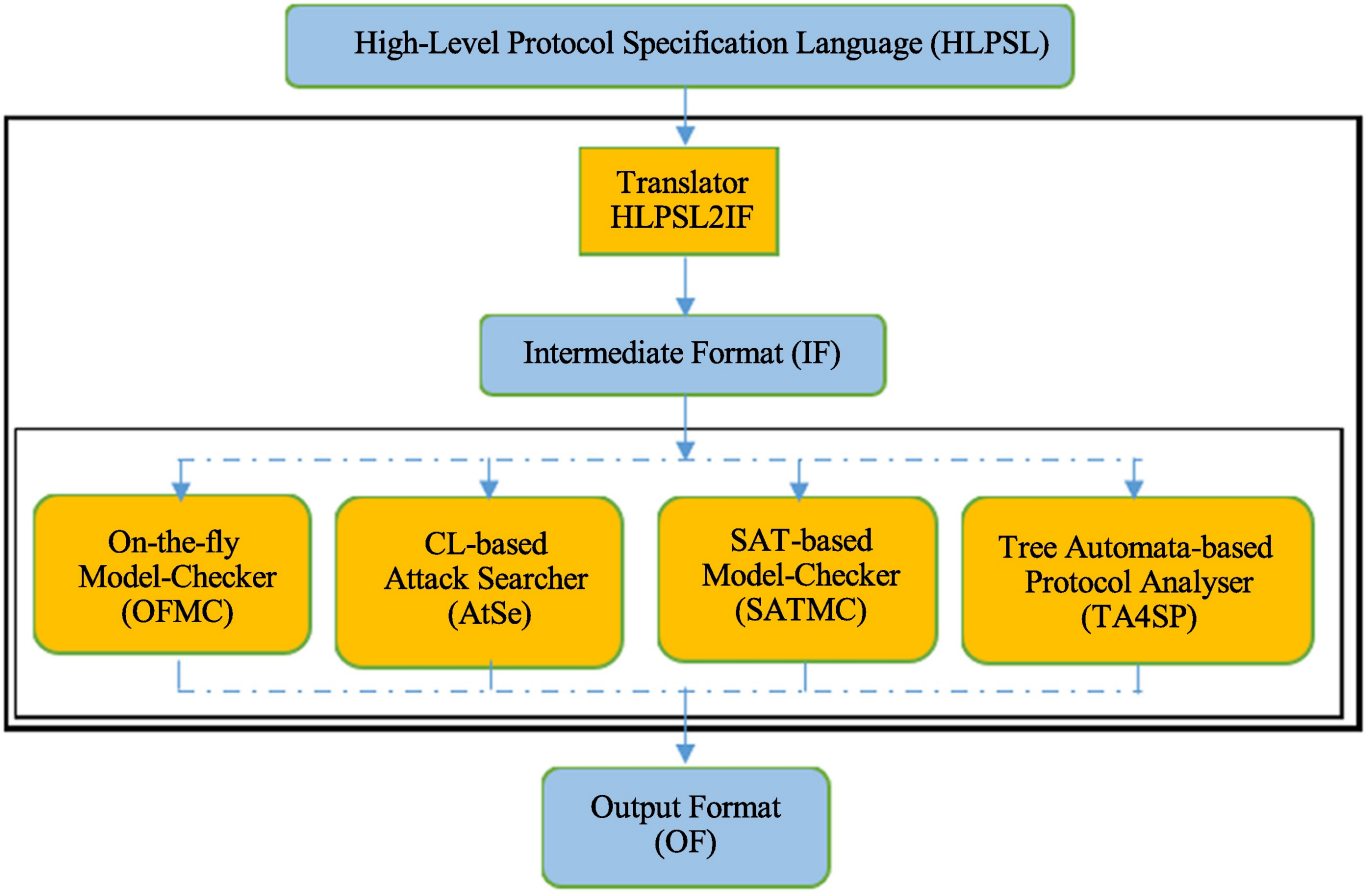
Architecture of the AVISPA tool.

AVISPA (Automated Validation of Internet Security Protocols and Applications) is a push-button software tool for the automated validation of Internet security-sensitive protocols and applications [44]. The AVISPA supports High Level Protocol Specification Language called as HLPSL and is usually used to provide the formal security verification of the simulated protocol. The simulation results in AVISPA can point out that whether proposed protocol is secure against the active and passive attacks. The architecture of the AVISPA tool is depicted in Fig 3 and its detailed introduction can be found in [44].

**Fig 4:**
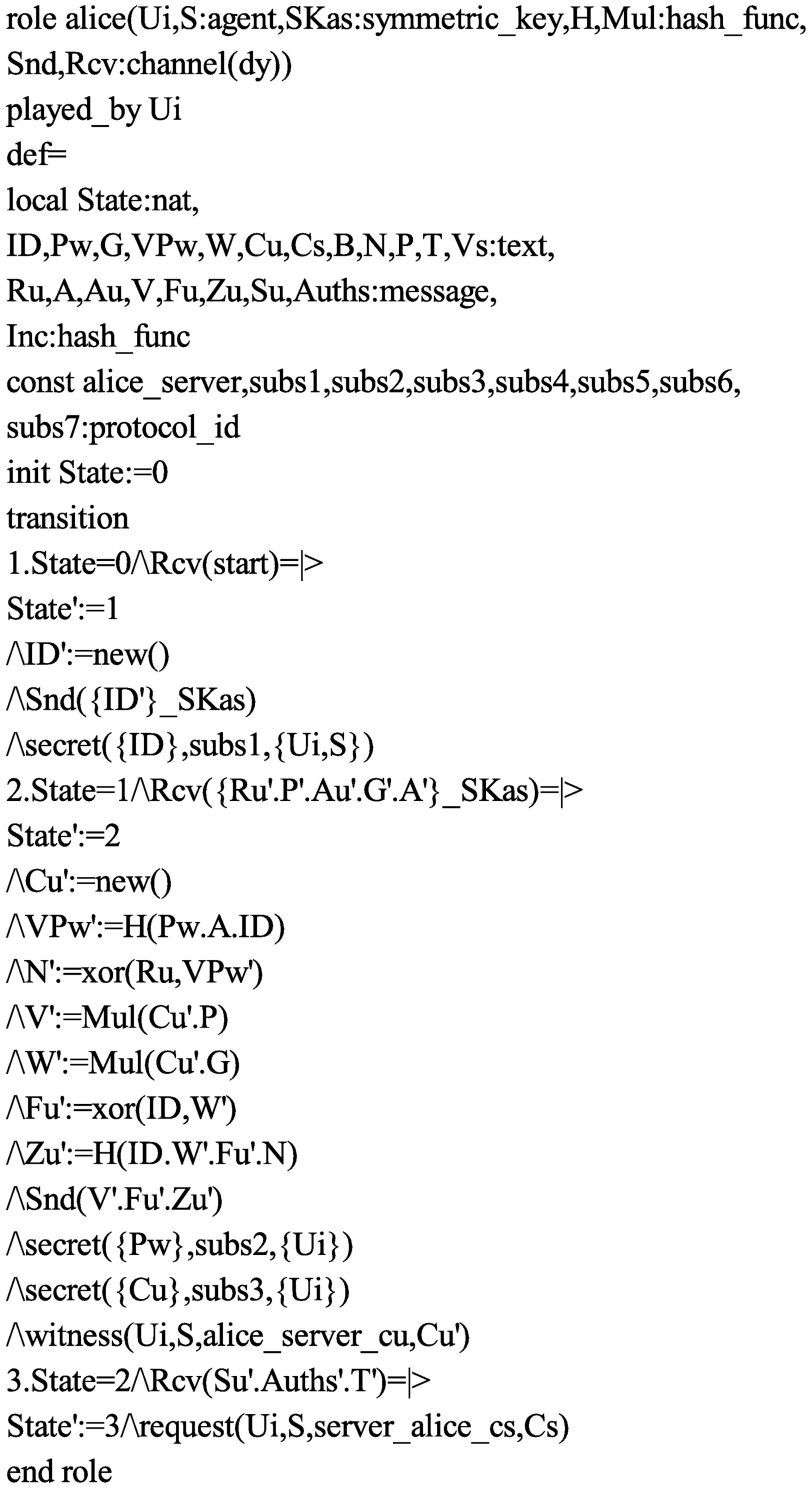
Role specification of *U*_*i*_ in HLPSL.

**Fig 5:**
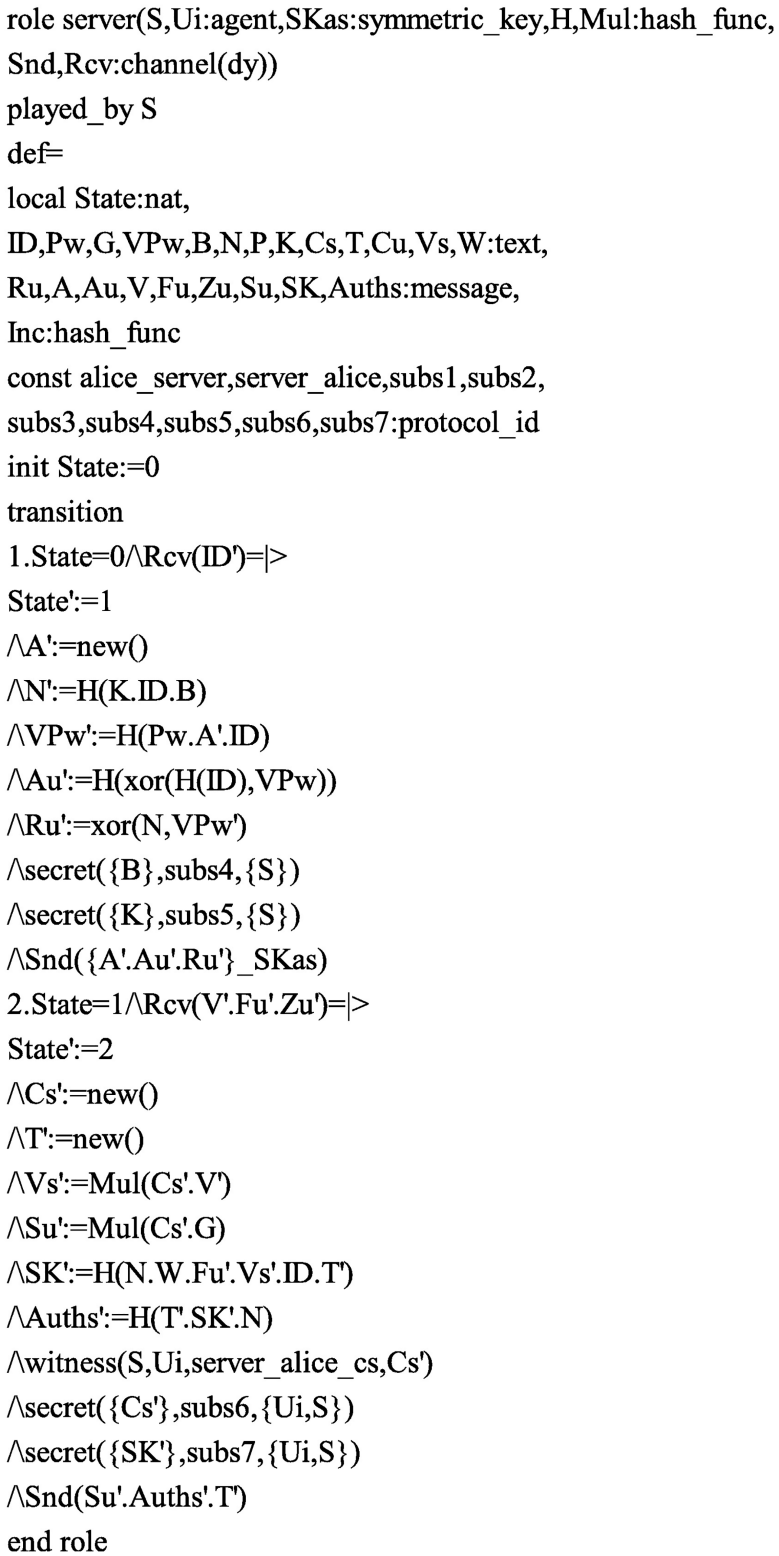
Role specification of *S* in HLPSL.

**Fig 6:**
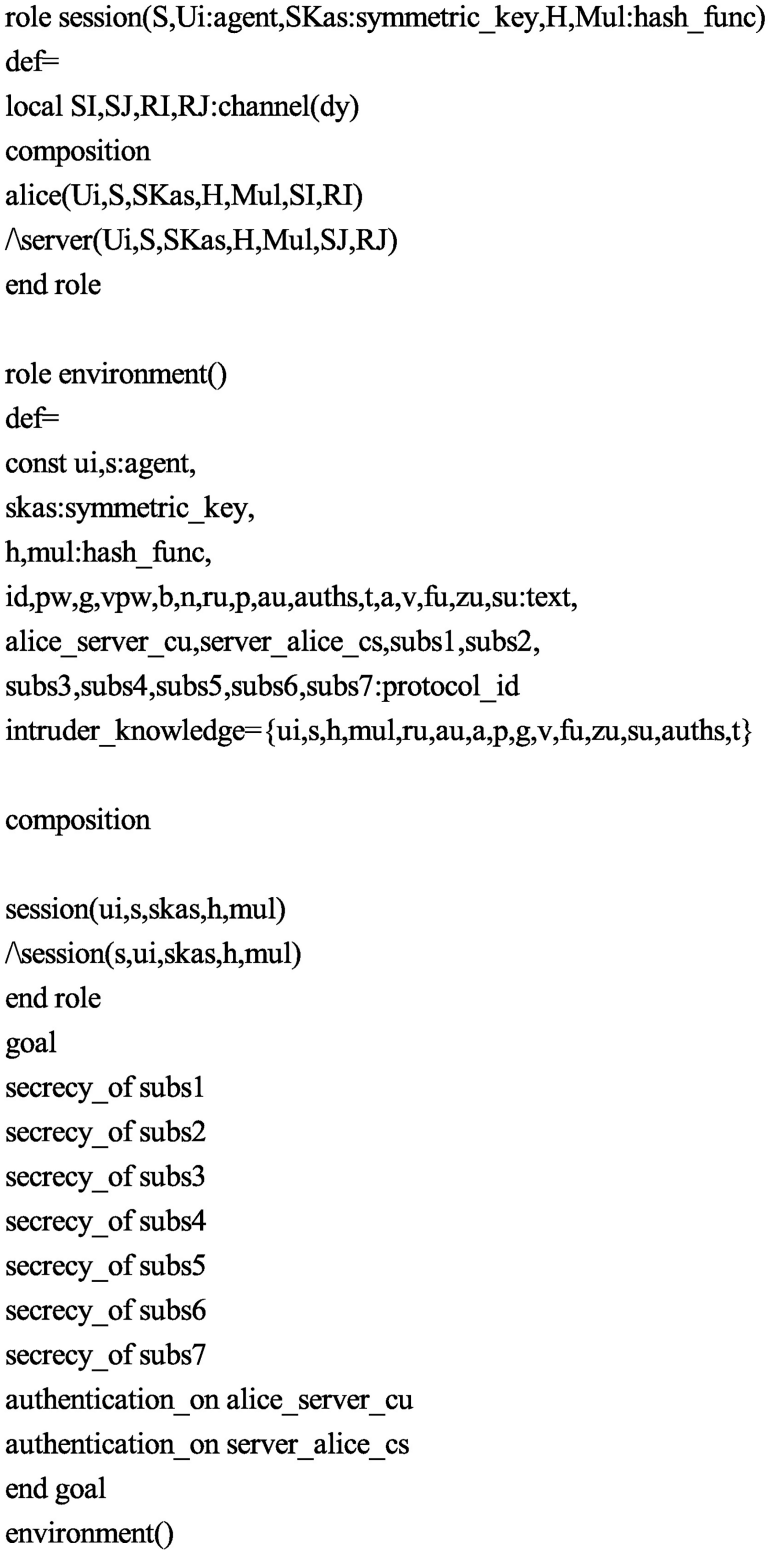
Role specification of the session, goal and environment in HLPSL.

**Fig 7:**
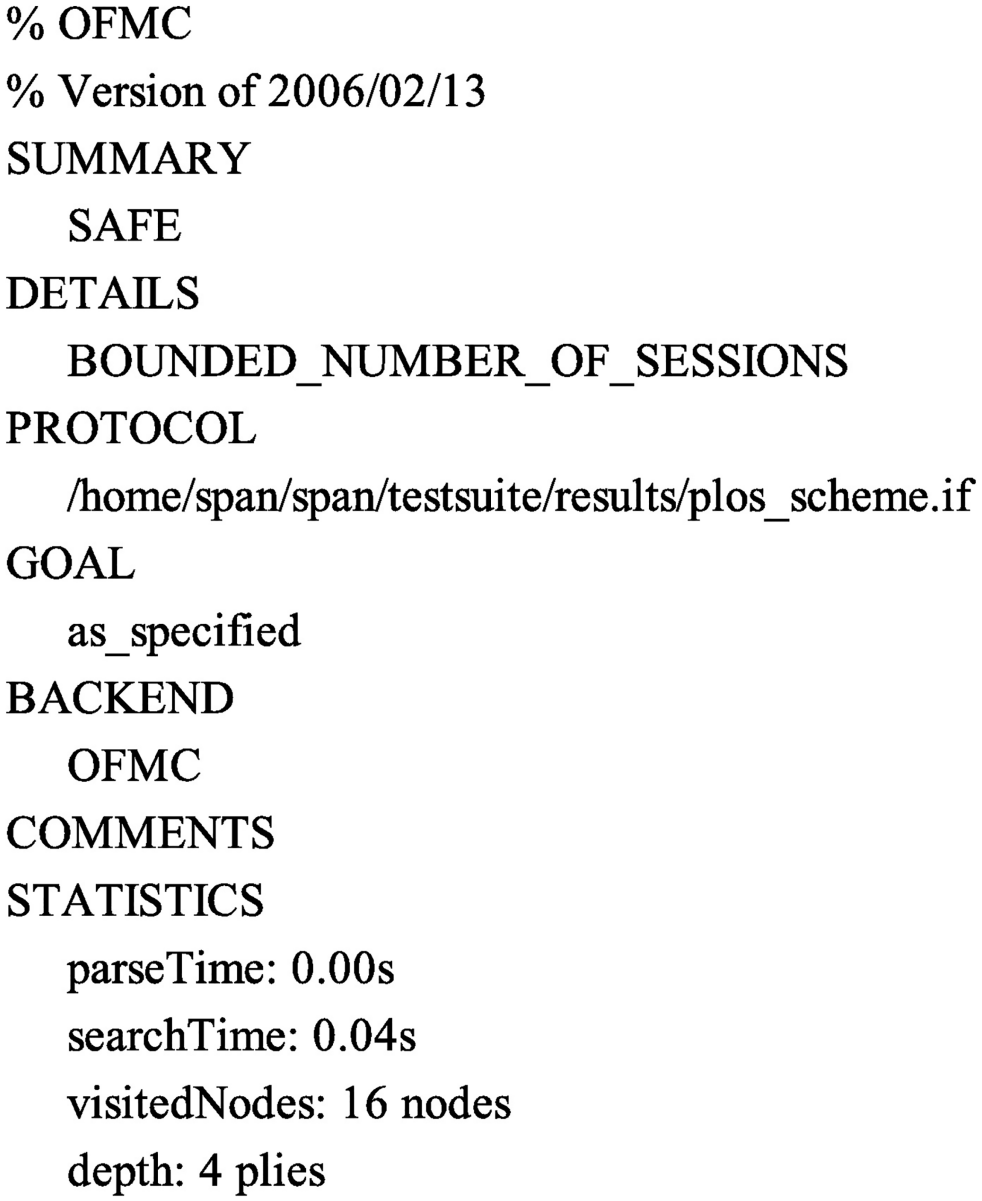
The simulation result using the OFMC backend.

**Fig 8:**
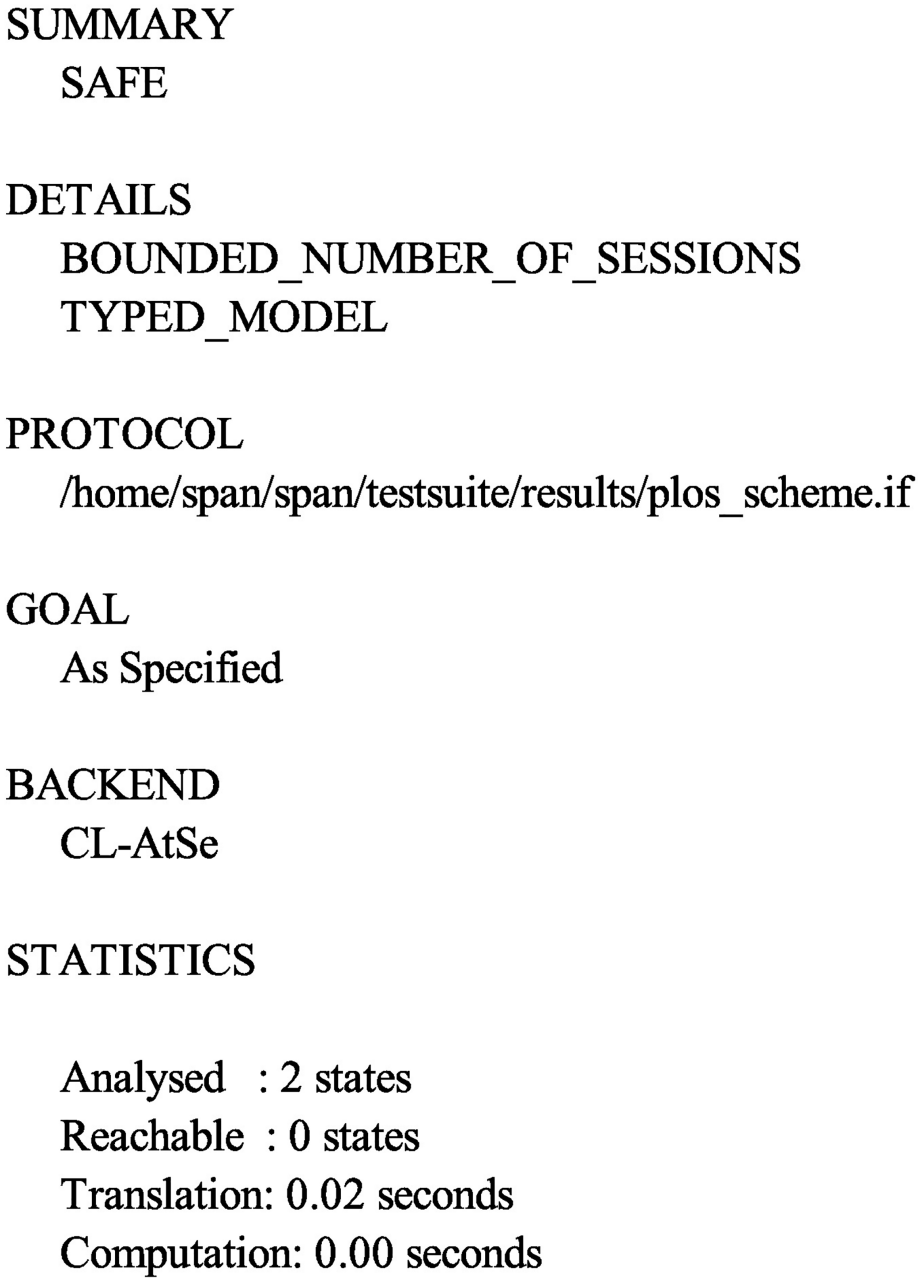
The simulation result using the CL-AtSe backend.

Accordingly, in order to test the security of the proposed protocol, we also use the AVISPA software tool to simulate it. Firstly, we translate the proposed protocol in HLPSL. The specifications for the roles for the user *U*_*i*_, the server *S*, the session, goal and environment in HLPSL are depicted in Figs 4, 5 and 6, respectively. Since only OFMC and CL-AtSe backends support the Diffie-Hellman and the bitwise exclusive-OR (XOR) operation, after execution through the OFMC and CL-AtSe backends, the simulation results ensure that our proposed protocol is SAFE against the active and passive attacks under the Dolev-Yao model [45]. The simulation results of the proposed scheme are provided in Figs 7 and 8.

## 8 Comparative analysis of performance

This section analyzes the performance of our proposed scheme by comparing it with Zhang et al.’s [27], Jiang et al.’s [34], Irshad et al.’s [31], Chaudhry et al.’s [38], Tu et al.’s [36], Zhang et al.’s [26], Farash’s [37] and Kumari et al.’s [39] schemes. Generally, in order to compare the computational complexity, we neglect the lightweight operations like exclusive-OR operation and string concatenation. We list some operations’s descriptions used in our paper as below:

- *T*_*pa*_: the time for performing an elliptic curve point addition operation.
- *T*_*pm*_: the time for performing a point multiplication operation.
- *T*_*me*_: the time for performing a modular exponentiation operation.
- *T*_*sed*_: the time for performing symmetric cryptography.
- *T*_*h*_: the time for performing a hash operation.

According to the experimental results performed as [46], *T*_*h*_, *T*_*pm*_, *T*_*pa*_ and *T*_*sed*_ take approximately 0.0023*ms*, 2.226*ms*, 0.0288*ms* and 0.0046*ms*, respectively. The above timings are obtained on a personal computer which has a Intel Pentium Dual CPU E2200 2.20GHz processor, 2048 MB of RAM and the Ubuntu 12.04.1 LTS 32bit operating system [46].

In this section, the comparative analysis is twofold as follows:

- Comparison of computational complexity (Table 2)
- Comparison of security features (Table 3)

**Table 2:** Comparison of computational complexity in login-authentication phase.

Scheme	User computations	Server computations	Total of computation overhead
Zhang et al. [27]	4*T*_*h*_ + 3*T*_*pm*_	4*T*_*h*_ + 4*T*_*pm*_	8*T*_*h*_ + 7*T*_*pm*_ ≈ 15.6004*ms*
Jiang et al. [34]	5*T*_*h*_ + 4*T*_*pm*_ + 1*T*_*pa*_	4*T*_*h*_ + 4*T*_*pm*_ + 1*T*_*pa*_	9*T*_*h*_ + 8*T*_*pm*_ + 2*T*_*pa*_ ≈ 17.8863*ms*
Irshad et al. [31]	6*T*_*h*_ + 4*T*_*pm*_	6*T*_*h*_ + 3*T*_*pm*_	12*T*_*h*_ + 7*T*_*pm*_ ≈ 15.6096*ms*
Chaudhry et al. [38]	5*T*_*h*_ + 3*T*_*pm*_ + 1*T*_*pa*_	5*T*_*h*_ + 3*T*_*pm*_ + 2*T*_*sed*_	10*T*_*h*_ + 6*T*_*pm*_ + 1*T*_*pa*_ + 2*T*_*sed*_ ≈ 13.417*ms*
Tu et al. [36]	4*T*_*h*_ + 3*T*_*pm*_ + 1*T*_*pa*_	4*T*_*h*_ + 3*T*_*pm*_	8*T*_*h*_ + 6*T*_*pm*_ + 1*T*_*pa*_ ≈ 13.4032*ms*
Zhang et al. [26]	6*T*_*h*_ + 1*T*_*pa*_ + 5*T*_*pm*_	4*T*_*h*_ + 2*T*_*pa*_ + 4*T*_*pm*_	10*T*_*h*_ + 3*T*_*pa*_ + 9*T*_*pm*_ ≈ 20.1434*ms*
Farash [37]	5*T*_*h*_ + 3*T*_*pm*_ + 1*T*_*pa*_	4*T*_*h*_ + 3*T*_*pm*_	9*T*_*h*_ + 6*T*_*pm*_ + 1*T*_*pa*_ ≈ 13.4055*ms*
Kumari et al. [39]	5*T*_*h*_ + 1*T*_*pa*_ + 3*T*_*pm*_	5*T*_*h*_ + 2*T*_*me*_	10*T*_*h*_ + 1*T*_*pa*_ + 5*T*_*pm*_ ≈ 11.1818*ms*
Ours	7*T*_*h*_ + 3*T*_*pm*_	5*T*_*h*_ + 3*T*_*pm*_	13*T*_*h*_ + 6*T*_*pm*_ ≈ 13.3859*ms*

**Table 3:** Comparison of security features.

Security features	Zhang et al. [27]	Jiang et al. [34]	Irshad et al. [31]	Chaudhry et al. [38]	Tu et al. [36]	Zhang et al. [26]	Farash [37]	Kumari et al. [39]	Ours
*F*_1_	No	No	Yes	No	No	No	No	Yes	Yes
*F*_2_	Yes	Yes	Yes	Yes	Yes	Yes	Yes	Yes	Yes
*F*_3_	No	No	No	No	No	No	No	No	Yes
*F*_4_	□	□	□	□	□	□	□	No	Yes
*F*_5_	Yes	Yes	No	Yes	No	□	Yes	Yes	Yes
*F*_6_	No	No	Yes	Yes	No	Yes	No	Yes	Yes
*F*_7_	Yes	No	No	Yes	No	Yes	No	Yes	Yes
*F*_8_	Yes	Yes	No	□	No	Yes	No	Yes	Yes
*F*_9_	Yes	Yes	Yes	Yes	No	Yes	Yes	Yes	Yes
*F*_10_	Yes	Yes	Yes	Yes	Yes	Yes	Yes	Yes	Yes
*F*_11_	Yes	Yes	Yes	Yes	Yes	Yes	Yes	No	Yes
*F*_12_	No	No	No	No	No	No	No	No	Yes

*F*_1_: Provides user anonymity and user un-traceability; *F*_2_: Resists privileged insider attack; *F*_3_: Provides pre-verification in the smart card; *F*_4_: Resists key-compromise impersonation attack; *F*_5_: Resists server impersonation attack; *F*_6_: Resists off/On-line password guessing attack; *F*_7_: Resists replay attack; *F*_8_: Resists session-specific temporary information attack; *F*_9_: Resists man-in-the-middle attack; *F*_10_: Provides mutual authentication; *F*_11_: Provides perfect forward secrecy; *F*_12_: Provides efficient password changing. “Yes” means the property is satisfied; “No” means the property is not satisfied and “▫” means the property is not discussed.

According to Table 2, the total computational costs of our proposed scheme in login and authentication phase is 13*T*_*h*_ + 6*T*_*pm*_ ≈ 13.3859*ms*. The results provide that the proposed scheme outperforms [26, 27, 31, 34, 36–38]. In comparison to Kumari et al. [39], our scheme has slightly more computational costs. However, it is an acceptable range under the trade-off of security and usability.

From Table 3, we observe that these proposals [26, 27, 31, 34, 36–39] lack some security ingredients and have more security problems than the proposed scheme. In Kumari et al.’s scheme [39], the authors declared that their protocol is secured against user impersonation attack, password guessing attack and session-specific temporary information attack applicable on Farash’s scheme [37]. On one hand, it is well known that perfect forward secrecy is a key security feature of key agreement scheme. Perfect forward secrecy ensures the security of the session key. On the other hand, key-compromise impersonation attack is also a fatal attack on SIP. If we have measures to resist this attack, why not to design such scheme? However, according to our observation, we find that Kumari et al.’s scheme [39] cannot provide the perfect forward secrecy and is vulnerable to key-compromise impersonation attack. Meanwhile, key-compromise impersonation attack is not considered by all schemes of Table 3, expect our scheme. Fortunately, we have taken effective measures to tackle key-compromise impersonation attack in our scheme, that is, the server stores random secret values *b* in its database. Besides, the proposed protocol utilizes the technique of “fuzzy-verifiers” [40] to resist off-line identity guessing attack and provides more security features, including pre-verification in the smart card and efficient password changing. Therefore, the proposed scheme not only address the security problems of Kumari et al.’s scheme [39] but also retains all their merits as depicted in Table 3. Although, our scheme employs a slightly complex elliptic curve point multiplication operation, but, as a trade-off, it can resist all known-attacks that are very important ingredients of the security of mutual authentication.

## 9 Conclusion

In this paper, we have provided a security analysis of Kumari et al.’s scheme [39] to prove that their scheme [39] is vulnerable to key-compromise impersonation attack and does not provide perfect forward secrecy, pre-verification in the smart card and efficient password changing. In order to remedy these limitations in Kumari et al.’s [39] scheme, we propose an enhanced authentication scheme with refined security. The proposed scheme inherits the merits of the Kumari et al.’s [39] scheme, resists the aforementioned attacks and provides more comprehensive security features with a slightly high computational cost than [39]. Additionally, the simulating results of the proposed protocol using AVISPA software infer that this proposed protocol is secure against active and passive attacks. Finally, in comparison with the previously proposed schemes, we conclude that the proposed protocol is more secure and effective to be implemented in real-life scenarios. Actually, many of the existing protocols can not be unconditional security. In order to enhance the security of the authentication protocol, a number of three-factor authentication protocols have been designed. Therefore, in our future work, we will design a more secure three-factor mutual authentication protocol based on smart cards to be implemented in many practical scenarios, such as: Internet of Things, Wireless Sensor Networks, Medical Care Systems, Vehicular Ad Hoc Networks, etc.

## Supporting information

S1 Fig Registration and authentication phase of our scheme. (EPS)

S2 Fig Architecture of the AVISPA tool. (EPS)

S3 Fig Role specification of *U*_*i*_ in HLPSL. (EPS)

S4 Fig Role specification of *S* in HLPSL. (EPS)

S5 Fig Role specification of the session, goal and environment in HLPSL. (EPS)

S6 Fig The simulation result using the OFMC backend. (EPS)

S7 Fig The simulation result using the CL-AtSe backend. (EPS)
